# Emulsifying activity of a biosurfactant produced by a marine bacterium

**DOI:** 10.1007/s13205-016-0494-7

**Published:** 2016-08-22

**Authors:** K. Abraham Peele, V. Ravi Teja Ch., Vidya P. Kodali

**Affiliations:** 1 Department of Biotechnology, Vignan’s University, Vadlamudi, Guntur Dist., Andhra Pradesh India; 2 Department of Biotechnology, Vikrama Simhapuri University, Nellore Dist., Andhra Pradesh India

**Keywords:** Bioemulsifier, Biodegradation, Bioremediation, Hydrocarbon, Marine microbiology

## Abstract

Biosurfactants produced by biofilm-forming bacteria have great applications in biotechnology, pharmaceutical, food engineering, bioremediation, and biohydrometallurgy industries. This study aimed to find out the bacteria that produce novel exopolymers (EPSs) which can find potential role in oil biodegradation. A screening procedure was performed to detect EPS-producing bacteria. The EPS producing isolate was identified as *Acinetobacter* species by 16S rDNA analysis. The polymer produced by the isolate has shown significant emulsification and surfactant activities, and the activities were compared to some of the commercial emulsifiers. The EPS has been partially characterized by FTIR analysis and has been proved to be a glycolipoprotein. This is one of the very few reports on *Acinetobacter* species producing EPS with surfactant properties.

## Introduction

Biosurfactants are surface-active and structurally diverse group molecules that are synthesized by the microbial cells. Most of the surfactants being used are chemically synthesized. There is an observable increase in the interest among the scientists on microbial emulsifiers because of their potential applications in environmental protection, low toxicity, high biodegradability, and high foaming capacity. Bioemulsifiers get accumulated at the interphase between the two immiscible phases by which they can reduce the surface tension, thereby resulting in the increased solubility and emulsification of the immiscible phases. Bioemulsifiers can convert the insoluble substrate into soluble substrates which can be utilized by the microorganisms for their metabolism (Rodrigues et al. 2006). Biosurfactants with such surface properties stood as good examples for enhanced oil recovery (EOR). Some of the biosurfactants are very effective, and they can reduce the surface tension of water from 72 dynes/cm to value range of 25–30 dynes/cm (Satpute et al. 2010). Lipopeptides belong to a class of biosurfactants that have shown remarkable surface active properties, viz., surplus crude oil recovery, food processing, de-emulsification, antimicrobial, antitumor, antiviral, and anti adhesive activities (Bodour et al. 2004). Biosurfactants have been shown dispersant activity by reducing the surface tension of oil–water interface effectively. Many bacteria like *Acinetobacter*, *Rhodococcus* species, and *Actinomycetes*, and other biofilm forming bacteria have been reported to produce biosurfactants. However, there is only less abundance of biosurfactant producing microorganisms that can be grown in natural environments than in contaminated environments. Growth pattern and biofilm formation may depend on various development factors, such as surface area, smoothness, flow velocity, and nutrients (Donlan and Costerton 2002). Hence, this study aims to isolate and molecular characterize an emulsifier producing bacterium from marine source.

## Materials and methods

### Isolation of EPS producer and biofilm assay

The marine water samples were collected from Bay of Bengal at Bapatla, Andhra Pradesh. Serial dilutions were performed for the screening of bacteria with the highest EPS producing ability. These marine isolates were grown using Nutrient broth (Hi media, Mumbai). Tube staining method with crystal violet is used to test the biofilm producing ability of the microbes (Abraham et al. 2012; Fabres-Klein et al. 2015). The highest EPS producer was screened for biosurfactant activity and was characterized biochemically. Identification of the strain was carried out by 16S r-DNA analyses.

### Staining and visualization of EPS with fluorescent labeled lectins

For the visualization of EPS by sample M, glass slides were immersed in bacterial suspension and kept in Petri dishes for 7 days at 37 °C for the biofilm formation, then the glass slide surface is covered with 50 μl DAPI solution for 15 min, and then the slide was washed with 1 ml PBS and distilled water. Treated slide was observed under fluorescence microscope (Carl Ziess) after drying (Pal and Paul 2013).

### Biosurfactant production and quantification

The biosurfactant production was studied in Luria broth (LB) by growing sample M for 14 days under submerged conditions at various pH and temperature conditions. The supernatant was collected by centrifugation, dried, lyophilized, and analyzed for biosurfactant production by measuring total carbohydrate, lipid, and protein contents (Dubois et al. 1951; Lowry et al. 1951; Kodali and Sen 2008). The critical micelle concentration (CMC) of biosurfactant was measured in PBS buffer using K6 force tensiometer (Kruss, Germany) (Hayder et al. 2014).

### Emulsifying potential of the biosurfactant

Emulsifying potential of the biosurfactant of sample M was found out against hydrocarbons such as (benzene and xylene), vegetable oils such as (olive and sunflower), and crude oils such as (kerosene, diesel, and petrol). All these hydrocarbons were of analytical grade (Fischer scientific, USA), and the rest of the oils were bought from local suppliers. The emulsifying activity was measured by combining equal volumes of biosurfactant solution (1 % w/v) and the hydrophobic substrates in 10-mm-diameter tubes, mixed on a vortex for 2 min and left to stand for 24, 96 and 168 h at 30 °C. Emulsifying activity was determined, and it is the percentage of the total height that was occupied by the emulsion after a time period of 24 h (Camacho-Chab et al. 2013).

### Thin-layer chromatography and FTIR analysis

The biosurfactant was dissolved in methanol and spotted on a 10 × 10 cm precoated silicagel GF 254. Samples will be prepared by the homogeneous dispersal of 1 mg of the biosurfactant sample, in pellets of potassium bromide, and now, these samples were subjected for FTIR analysis. IR spectra were collected over the range of 400–4000 cm^−1^ and with a resolution of 4 cm^−1^ (Das et al. 2008; Thompson et al. 1994).

## 16S rDNA sequence analysis

The 16S rDNA analysis of the sample M was analyzed. DNA purification kit (PureFast^®^ Bacterial Genomic DNA kit), 16S Bac specific primer-forward (10 pico-moles/μL), and 16S Bac specific primer-reverse (10 pico-moles/μL) were used to amplify the 16S rDNA gene by PCR. The PCR mixture contained 50-μL final volume; 25 μL of master mix contains 10X Taq buffer, 2 mM MgCl_2_, 0.4 mM dNTPs mix, 2U Proofreading Taq DNA polymerase, and 1 μL genomic DNA. The DNA amplification was performed using Eppendorf PCR, System. The PCR program was as follows: 94 °C for 1 min, 60 °C for 1 min, and 72 °C for 1 min for 30 cycles each of 6 min at 72 °C. The PCR products were visualized under UV light after electrophoresis on a 2 % (w/v) agarose gel containing ethidium bromide. The PCR product was sent to Helini Biomolecules for sequence analysis. The nucleotide sequence of the 16S rDNA genes (about 1400 nucleotides each) was aligned using CLUSTAL W program (Thompson et al. 1994).

## Results and discussion

### Isolation and screening of EPS producer

**Fig. 1:**
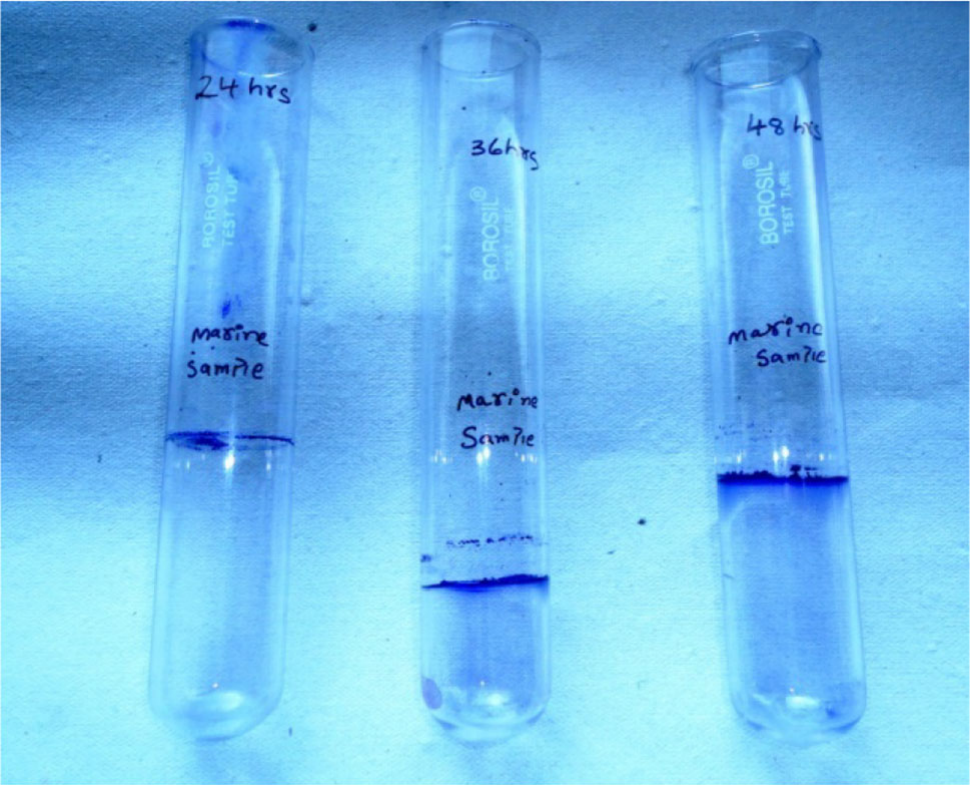
Biofilm assay by crystal violet staining

From six isolates, one isolate was selected based on its highest EPS (Biofilm) producing ability (Fig. 1), named sample M, and was characterized biochemically. The isolate was positive for catalase, laccase, and lactase tests, and negative for starch hydrolysis, methyl red and Voges-Proskauer, indole production, utilization of citrate, urease production, and hydrogen sulfide production tests. In drop collapsing test, a flat drop and, in oil displacement method, a clear zone were observed (Andreoni et al. 2000; Rodrigues et al. 2006).

### Staining and visualization of EPS with fluorescently labeled lectins

**Fig. 2:**
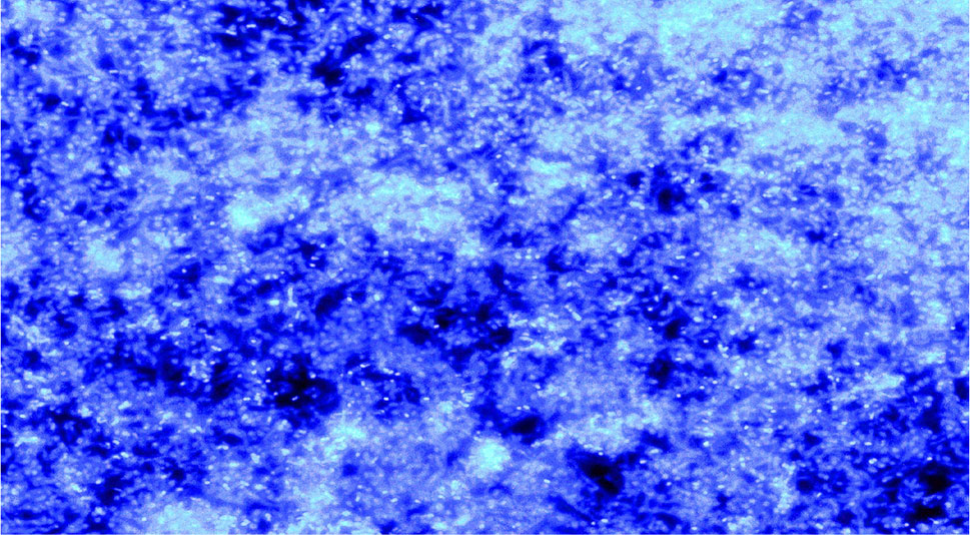
Visualization of bacterial cells and biofilm EPS by staining with DAPI by fluorescence microscopy

The biofilm of sample M was stained with DAPI and visualized by fluorescence microscope (Fig. 2) DAPI stains the cells and extracellular matrix by passing through the cell membrane and allows the microscopic detection of the biofilm.

### Biosurfactant production and quantification

**Fig. 3:**
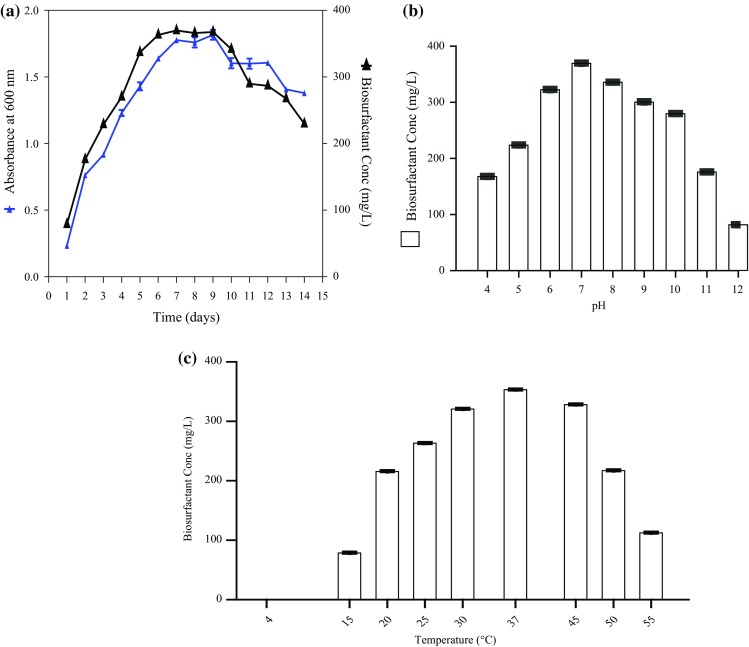
**a** Time course (growth kinetics) of sample M produced biosurfactant. **b** Effect of pH on biosurfactant production by sample M. **c** Effect of temperature on biosurfactant production by sample M

**Fig. 4:**
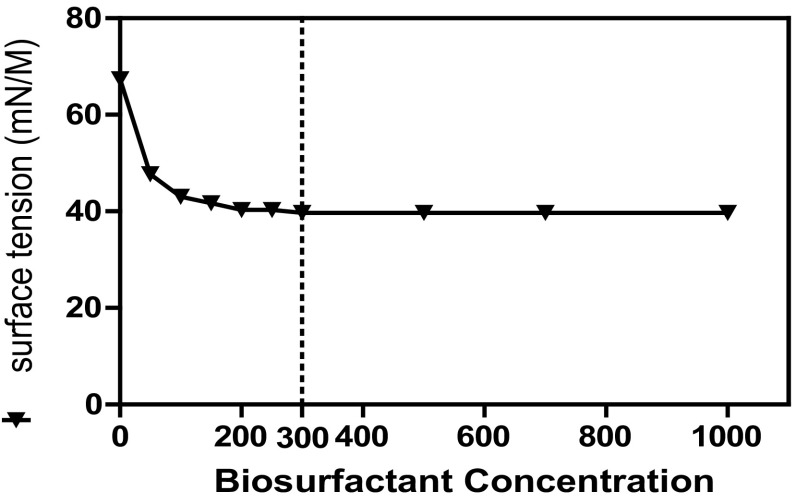
Critical micelle concentration of bioemulsifier produced by sample M

The biosurfactant produced by sample M was extracted and dissolved in 1 ml of demineralized water. The total carbohydrate and protein concentrations were observed to be 310 and 150 µg/ml, respectively. Sample M showed maximum biosurfactant production at pH 7.0 and 37 °C (Fig. 3). The addition of biosurfactant at the concentration of 300 mg/l to PBS buffer and CMC reduced the surface tension value to 39 and of 39 mN/m, respectively (Fig. 4) (Kokare et al. 2007; Khopade et al. 2012).

### Emulsifying potential of the biosurfactant

**Fig. 5:**
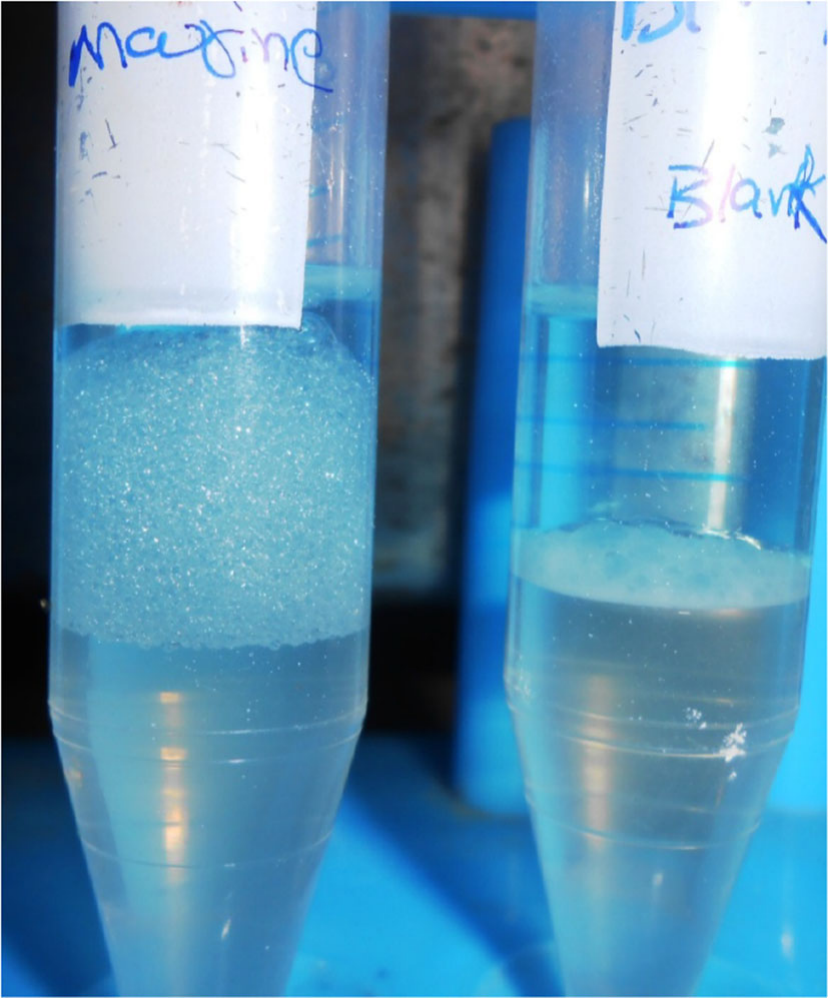
Emulsification potential of bioemulsifier in kerosene (1 % w/v) at 24 h

The emulsifying activity of biosurfactant from sample M was tested against different hydrocarbon substrates. The emulsifying activity of biosurfactant was evaluated after 24, 96, and 168 h. The emulsions found to be stable even after 7 days. Synthetic surfactants Triton X-100 and Tween 20 were more efficient than biosurfactant, and the two natural biopolymers showed emulsification activities of 100 % against oils after 168 h (Table 1). Biosurfactant showed stronger emulsifying activities than gum arabic, and the emulsifying activity of biosurfactant was dependent on the type of substrate (Camacho-Chab et al. 2013). These results indicated that biosurfactant of sample M was a good emulsifier (Fig. 5).

**Table 1:** Emulsifying activity of biosurfactant, synthetic surfactants, and bioemulsifiers on various hydrophobic substrates after 168 h of incubation at 30 °C

Hydrophobic substrate	Emulsifying activity of Biosurfactant	Emulsifying activity of Synthetic surfactants		Emulsifying activity of Biopolymers	
		Tween 20	Triton X-100	Gum arabic	Xanthan gum
Xylene	74 ± 0.91	94 ± 0.72	95 ± 0.96	78 ± 1.16	78 ± 1.19
Benzene	67 ± 1.42	94 ± 0.97	68 ± 0.70	82 ± 2.13	72 ± 1.35
Toluene	71 ± 0.97	82 ± 0.24	81 ± 1.93	70 ± 1.85	70 ± 3
Diesel	76 ± 1.23	73 ± 0.37	68 ± 2	89 ± 0.48	89 ± 1.2
Petrol	78 ± 0.78	68 ± 2.2	72 ± 0.67	88 ± 0.84	87 ± 3
Kerosene	67 ± 0.82	67 ± 1.46	69 ± 0.25	78 ± 0.87	75 ± 0.89
Motor oil	81 ± 0.94	69 ± 1.32	67 ± 0.49	93 ± 0.71	91 ± 0.75
Olive oil	73 ± 2.05	100	100	99 ± 0.49	83 ± 0.84
Sunflower oil	76 ± 0.89	100	98 ± 0.82	98 ± 0.2	86 ± 0.41

### Thin-layer chromatography and FTIR analysis

**Fig. 6:**
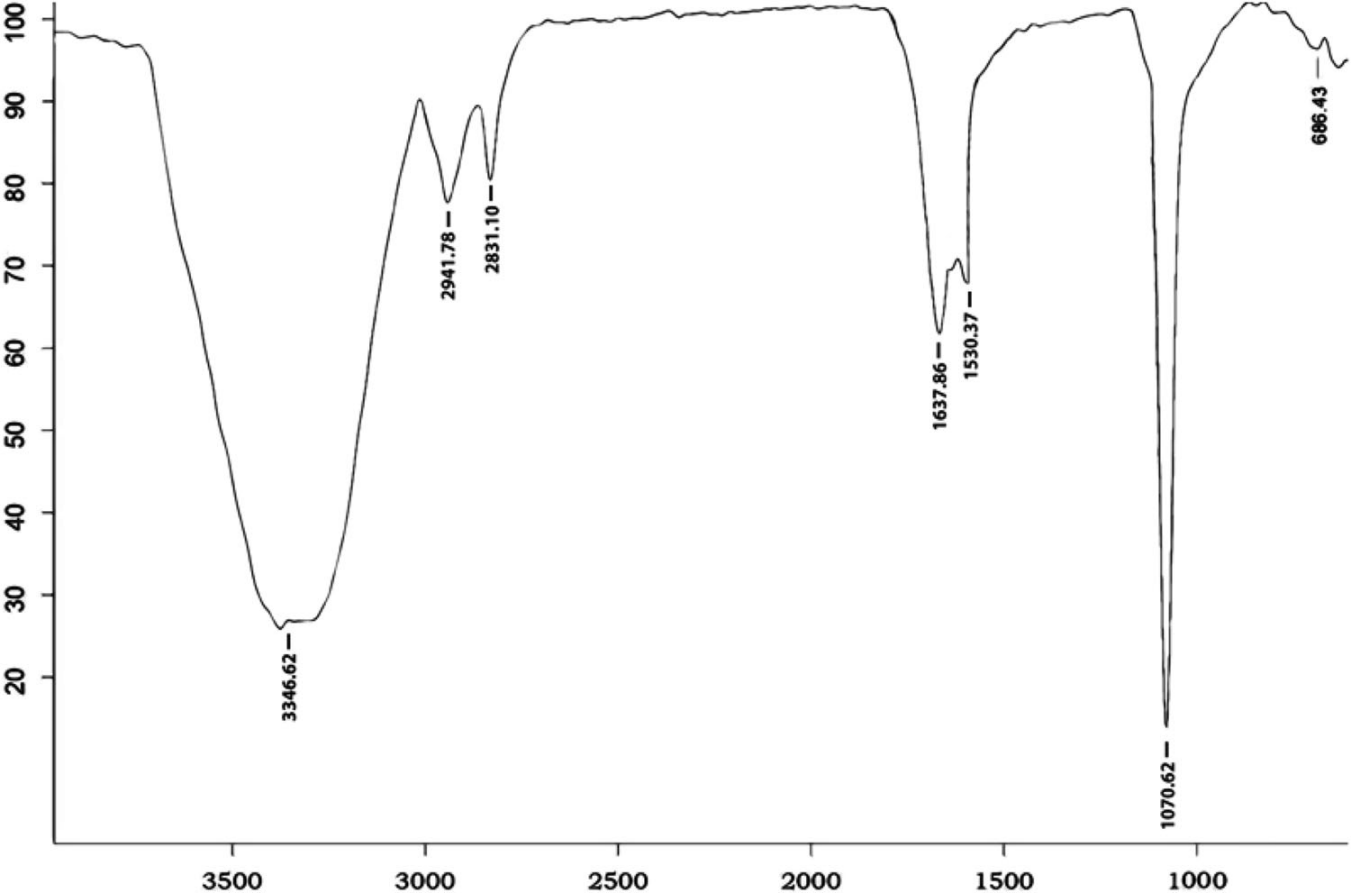
FTIR analysis of biosurfactant produced by sample M

**Fig. 7:**
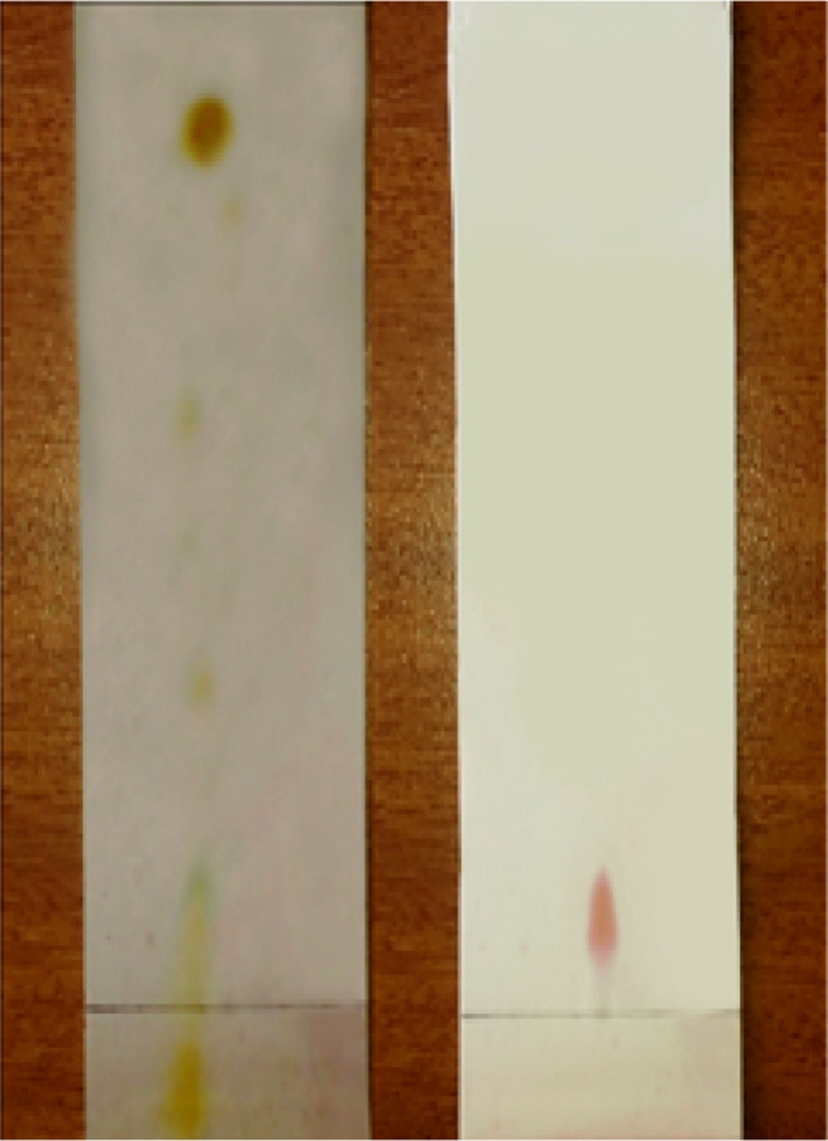
TLC analysis of biosurfactant produced by sample M

The ninhydrin solution and anthrone reagent developed plates showed the confirmation of peptides and lipids as red and yellow spots giving the preliminary analysis of the biosurfactant extracted from sample M are lipopeptide in nature. The FTIR spectrum of the biosurfactant produced by sample M showed a broad O–H stretching band at 3346 cm^−1^ and at an intense band at 1070 cm^−1^ which is an indicative for typical of carbohydrates. In addition, bands at 1637 and 1530 cm^−1^ indicating (Fig. 6) the presence of lipids, shown by the peak at 2941 cm^−1^ in the FT-IR analysis represent the asymmetric stretch (C–H) of –CH_2_ groups combined with that of –CH_3_ groups in lipids. The presence of lipid content carbohydrates and proteins suggests that biosurfactant of sample M was a class of glycolipoprotein (Fig. 7) (Bergey and Holt 1994).

## 16S rDNA sequence analysis

**Fig. 8:**
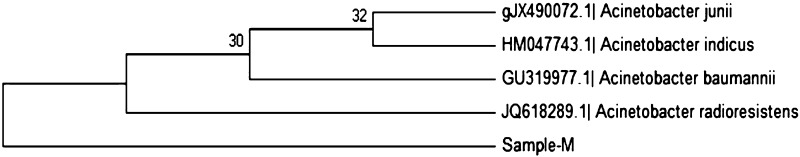
Phylogenetic tree based on 16S rDNA gene sequences, showing the positions of strain sample M (*Acinetobacter M6*, Accession no: KR559749) relative to all known *Acinetobacter* species. Accession numbers of 16S rDNA gene sequences of reference organisms are also shown

The phylogenetic position of sample M based on its 16S rDNA gene confirmed that its closest relative was *Acinetobacter* genus (Fig. 8). However, definitive species identification of this bacterial isolate requires an approach including biochemical, physiological, and nucleic acid-based methods (Stackebrandt and Ebers 2006).

### Statistical studies

All experiments were conducted in triplicate and analyzed with ANOVA, *t* test using Graph Pad Prism 5 software. Results represent standard error mean.

## Conclusion

From the above observation, it was concluded that our marine isolate, sample-M which was identified as *Acinetobacter* genus and was submitted to GenBank as *Acinetobacter* M6. The isolate produced a surfactant which was composed of glycolipoprotein. The experimental results showed that the biosurfactant has good surface active properties which could be developed into a promising molecule for industrial and environmental applications. Maximum biosurfactant production was observed in 7 days of incubation. The functional stability of this bioemulsifier was retained for long periods of time. This study extends a great knowledge on marine bacteria that produce biopolymers.

## Abbreviations

EPS: Exopolysaccharide
FTIR: Fourier transform infrared spectroscopy
PBS: Phosphate buffer saline
DAPI: 4′, 6-Diamidino-2-phenylindole
